# Measuring multiple-source based academic writing self-efficacy

**DOI:** 10.3389/fpsyg.2023.1212567

**Published:** 2023-07-13

**Authors:** Ivar Bråten, Ymkje E. Haverkamp, Natalia Latini, Helge I. Strømsø

**Affiliations:** Department of Education, University of Oslo, Oslo, Norway

**Keywords:** multiple-source based writing, synthesis writing, writing motivation, writing self-efficacy, measurement

## Abstract

Although writing self-efficacy has been a productive line of research for several decades, no prior writing self-efficacy measure has focused on students’ self-efficacy for integrating information across multiple sources when producing an academic text. To fill this gap in existing research on the measurement of writing motivation, we designed a measure targeting the extent to which students are confident that they can write an academic text that integrates content from several different sources. In a study with Norwegian undergraduate students (*n* = 136), this measure, which we called the Multiple-Source based Academic Self-Efficacy Scale (MAWSES), was validated by means of confirmatory factor analysis and relationships between the resulting unitary construct and other relevant constructs. The findings provided evidence concerning the reliability and validity of the MAWSES. In future research, this measure could be included as an independent variable to predict processes and products of multiple-source based, integrated academic writing, as a moderator or mediator of effects in writing intervention research, or as an outcome variable in its own right.

## Introduction

In higher education, writing is typically a multiple-source based activity in which students write about information gathered from a set of diverse sources on the same topic, issue, or phenomenon (Sonia et al., 2023). As these sources often present complementary (information across different sources is part of a larger whole not specified in any single source) or conflicting information, student writers are tasked with synthesizing or integrating information across different perspectives and arguments to demonstrate their writing competence or communicate their understanding. Such multiple-source based, integrated academic writing tasks have been found to represent a formidable challenge across educational levels that may require particular instructional interventions (Mateos et al., 2018; Weston-Sementelli et al., 2018; Granado-Peinado et al., 2019; Du and List, 2020; Kiili et al., 2020; Marttunen and Kiili, 2022; Barzilai et al., 2023; Kullberg et al., 2023; Vandermeulen et al., 2023a). As such, they can also be assumed to require considerable motivation on the part of the students, not least with respect to their confidence in their ability to successfully complete such tasks (i.e., their self-efficacy beliefs; Bandura, 1997). However, although writing self-efficacy has been a productive line of writing motivation research for several decades (for reviews, see Klassen, 2002; Bruning and Kauffman, 2016; Abdel Latif, 2021), no prior writing self-efficacy measure has been created that targets this specific writing task (Abdel Latif, 2021). We therefore created a process-focused, task-specific writing self-efficacy measure focused on the process of integrating information across multiple sources when completing the task of composing an academic text. In the current study, we performed a preliminary validation of this measure, analyzing the structure of the scores in addition to relationships between these scores and a range of variables considered relevant based on theories of writing (Hayes, 1996; MacArthur and Graham, 2016; McNamara and Allen, 2018) and prior research on writing motivation (Abdel Latif, 2021). Before we further specify the research questions that guided our study, we briefly discuss the role of motivation within theories of writing, conceptualizations and relevant research on multiple-source based writing, and prior research on writing self-efficacy and its measurement.

### Writing and motivation

In Flower and Hayes’ (1981) and Hayes and Flower’s (1980) classic cognitive process theory of writing, motivation was only represented as motivational cues in the task environment. However, when Hayes (1996) substantially revised this theory, motivation was featured as an important individual difference factor in writing, referring to writers’ goals, predispositions, beliefs and attitudes, and cost/benefit estimates. Further, motivation was assumed to be bidirectionally related to the task environment, as well as to writers’ text interpretation (i.e., reading comprehension), working memory and executive functions, and knowledge. Hayes (1996) did not specify how writers’ motivation could be assumed to draw upon and be influenced by environmental and cognitive factors, however. Other relevant individual difference factors, such as gender and language background (Abdel Latif, 2021), were also not considered in relation to motivation within this theoretical framework.

Another influential model of writing that highlights the importance of motivation is Zimmerman and Risemberg’s (1997) model of self-regulated writing. Based on Bandura’s (1986) social-cognitive theory, Zimmerman and Risemberg’s (1997) model describes how proficient writers monitor and regulate their behavior, cognition, and environment when completing writing tasks, with motivation for such self-regulated writing essentially stemming from writers’ perceived self-efficacy, that is, their perceived ability to perform the actions required to complete specific writing tasks. Further, the relationship between self-efficacy on the one hand and self-regulated writing and writing performance on the other was regarded as reciprocal, with self-efficacy not only influencing but also being influenced by writers’ self-regulation and performance (Zimmerman and Risemberg, 1997).

The application of the model of domain learning (Alexander, 1997, 2004) to the domain of writing (MacArthur and Graham, 2016) highlights how the motivational construct of interest interacts with writers’ strategies and knowledge, with more proficient writers characterized by higher individual interest in writing, the use of deeper level writing strategies (knowledge transformation), and more principled knowledge about writing and the processes of writing.

Finally, the writer(s)-within-community (WWC) model of writing by Graham (2018) presents a broader, more nuanced view on writing motivation. As such, it draws on a range of motivational theories, including expectancy-value theory (Wigfield and Eccles, 2000), self-efficacy theory (Bandura, 1997), self-determination theory (Deci and Ryan, 2000), goal-orientation theory (Elliot, 1999), and attribution theory (Weiner, 2005). Like Hayes’s (1996) model, the WWC model describes motivational constructs as interacting with writers’ working memory and executive functions.

In summary, although motivational constructs have been featured within several theoretical accounts of writing, none of these frameworks have addressed motivation for writing from multiple sources, in particular. In the following, we turn to this educationally relevant writing task and some crucial processes involved in that task.

### Multiple-source based academic writing

During the last decades, multiple-source based academic writing has been addressed by reading comprehension researchers focusing on multiple text comprehension and by writing researchers focusing on synthesis writing. Within the area of multiple text comprehension (also termed multiple document literacy; Bråten and Strømsø, 2010), process models have focused on purposeful literacy tasks in which students’ read multiple sources to construct an integrated understanding of a topic and subsequently communicate their understanding in the form of a written task product (Rouet and Britt, 2011; Britt et al., 2018; List and Alexander, 2019). In these models, the main emphasis has been on reading rather than writing, that is, on integrated understanding conceptualized as a coherent mental representation of the content included in different texts (Perfetti et al., 1999). That is, although writing tasks have quite often been used as post-reading assessment tools targeting multiple text comprehension in this area of research (Barzilai et al., 2018; Primor and Katzir, 2018), it seems fair to say that the attention to writing *per se* has been rather modest (McNamara and Allen, 2018; McCarthy et al., 2022). Accordingly, the lack of integration commonly observed in students’ writing task products (e.g., Anmarkrud et al., 2014; Du and List, 2020; Kiili et al., 2020; Kullberg et al., 2023) has typically been interpreted as an issue related to reading comprehension rather than writing competence within multiple text comprehension (McNamara and Allen, 2018; McCarthy et al., 2022). That said, in a recent study, McCarthy et al. (2022) demonstrated that students’ writing ability may be a unique predictor of their multiple text comprehension when the latter was assessed with a multiple-source based writing task. No attention was devoted to writing motivation in that study, however. A more direct focus on multiple-source based writing has been implemented by writing researchers primarily interested in synthesis writing (e.g., Segev-Miller, 2007; Solé et al., 2013; Mateos et al., 2018; Granado-Peinado et al., 2019; Vandermeulen et al., 2020b). Synthesis writing can be defined as source-based writing directed toward synthesizing information from different sources to compose a new text that can be understood by people without access to the original source materials (Vandermeulen et al., 2023b). This line of research has described how writers select, organize, and connect source information in order to produce a new discourse that is both loyal and transformative in relation to the sources (Spivey and King, 1989; Segev-Miller, 2007). Further, it has highlighted the recursive nature of reading and writing when writing synthesis text, with more adaptive switching between processes of reading and writing (e.g., reading and comprehending the sources, writing the synthesis text, reading and evaluating the synthesis text, and revising the synthesis text) characterizing more proficient synthesis writers (Solé et al., 2013; Vandermeulen, et al., 2020a,b). Individual difference variables addressed by synthesis writing researchers include educational level, reading comprehension skills, writing skills, reflection, prior knowledge, and topic interest (Spivey and King, 1989; Solé et al., 2013; Van Steendam et al., 2022; Castells et al., 2023). To the best of our knowledge, writing motivation has not been included in prior research on synthesis writing, however.

Taken together, research within multiple text comprehension and synthesis writing has emphasized the importance of integrating content across diverse sources in order to produce a new, cohesive, and understandable text. This may involve explaining, relating (e.g., comparing and contrasting), and reconciling different or opposing views on the topic discussed across the source texts, thereby providing readers with a credible overview of the topic in question. Needless to say, this is a cognitively demanding task that may require not only skill but also considerable will (i.e., motivation) on the part of the writers.

### Writing self-efficacy

Given the plethora of studies on the antecedents and consequences of students’ perceived self-efficacy following Bandura’s (1977) initial discussion of the construct, it is no wonder that researchers in the domain of writing quite soon began to target student writers’ confidence in their ability to perform specific writing tasks. Taken together, research on writing self-efficacy conducted over nearly four decades has strongly indicated that a positive relationship exists between students’ self-efficacy and their writing performance (Klassen, 2002; Bruning and Kauffman, 2016; Abdel Latif, 2021). However, findings regarding relationships between writing self-efficacy and a range of relevant individual difference variables have been less consistent.

Several studies have indicated higher self-efficacy for writing among females than among males (e.g., Hidi et al., 2002; Andrade et al., 2009). However, there are also some indications that such gender-related differences may be reduced and even reversed at higher educational levels (Abdel Latif, 2021), and that any differences in this regard may be related to gender orientation or gender identification rather than to gender *per se* (Pajares and Valiante, 2001).

With respect to language background, there is a general lack of research on the potential relationship between this variable and writing self-efficacy. To the extent that students who have another language background than the majority language perceive their own language ability to be problematic, it seems reasonable to expect that their self-efficacy for writing in the majority language could be lower than that of language majority students, however (Abdel Latif, 2021).

With respect to educational level, writing self-efficacy has been found to decline as students move beyond elementary school (Pajares and Valiante, 1999; Pajares et al., 2007a) but not necessarily when they move into and through the high school grades (Shell et al., 1995; Pajares et al., 2007b). Besides, prior research has hardly addressed potential differences in writing self-efficacy between students at different levels of postsecondary education, with more extensive study experience beyond high school possibly leading to higher writing self-efficacy (Mitchell et al., 2021).

In accordance with Bandura’s (1997) theory of self-efficacy, previous mastery experiences with writing (i.e., writing achievement) has been shown to be a strong predictor of students’ writing self-efficacy (Pajares et al., 2007b). However, few studies have so far compared the contribution of students’ previous writing achievement to their writing self-efficacy with that of other relevant predictors.

Finally, there seems to be a general lack of research on relationships between writing self-efficay and cognitive variables such as reading comprehension, working memory, and executive functions. Thus, although relationships between writing motivation and cognitive variables have been highlighted within cognitive perspectives on writing, including Hayes’s (1996) influential model, these cognitive variables (i.e., reading comprehension, working memory, and executive functions) have mainly been studied in relation to writing performance, not writing motivation (MacArthur and Graham, 2016; McNamara and Allen, 2018; Limbo and Olive, 2021). However, given that these cognitive variables may be linked to students’ mastery experiences with writing (McNamara and Allen, 2018), it seems reasonable to expect that they could be positively related to their writing self-efficacy as well. In particular, reading comprehension at the level of situation model construction (Kintsch, 1988), that is, inferential reading comprehension, seems important in this context. Moreover, working memory, which refers to a processing resource with limited capacity involved in the storage of information while simultaneously manipulating information for brief periods of time (Baddeley and Logie, 1999; Alloway, 2009; Swanson and Alloway, 2012), needs to be further studied in relation to writing self-efficacy. The same is true for executive functions, which can be defined as a set of separate yet related cognitive mechanisms involved in the regulation of behavior and cognition during the performance of challenging tasks (Miyake et al., 2000; Miyake and Friedman, 2012).

It also seems likely that some inconsistencies in research on writing self-efficacy in relation to other variables are due to differences in the way this construct has been measured across studies. In his comprehensive review of writing motivation measures, Abdel Latif (2021) noted that 21 different writing self-efficacy measures had been published and used since 1984, including unidimensional as well as multidimensional measures. As an example of an early unidimensional measure, Graham et al. (1993) used seven items to assess students’ perceived self-efficacy for performing basic composing processes related to planning, translating, and reviewing (Flower and Hayes, 1981). More recent multidimensional writing self-efficacy measures include Bruning et al.’s (2013) 16-item measure focusing on the three dimensions of self-efficacy for generating ideas, mastering writing conventions (mechanics, syntax), and self-regulating the writing process, and MacArthur et al.’s (2016) 18-item measure focusing on the three dimensions of self-efficacy for performing different writing tasks (e.g., introduction, summary, and conclusion writing), using strategies for planning, organizing, and revising text, and self-regulating writing by evaluating progress, managing time, and avoiding distractions.

Despite the merits of these previous measures of writing self-efficacy, we contend that a specific measure of self-efficay for multiple-source based writing in an academic task context may fill an important gap in the measurement literature. Crucial to our argument is the view shared by scholars in multiple document literacy and synthesis writing that integrating information across multiple sources is a critical process in academic writing (e.g., Rouet and Britt, 2011; Vandermeulen et al., 2023b). Gaining understanding about students’ perceived self-efficacy for multiple-source integration when composing academic text therefore seems like an important agenda for writing motivation research.

### The present study

In summary, theories of writing have included writing motivation as an important individual difference factor (Hayes, 1996; Zimmerman and Risemberg, 1997; Graham, 2018). Among the motivation constructs that have been addressed by writing researchers, writing self-efficacy holds a unique position (Klassen, 2002; Bruning and Kauffman, 2016; Abdel Latif, 2021). However, among the many measures developed and used to gauge this construct, none has focused on perceived self-efficacy for multiple-source based, integrated academic writing (Abdel Latif, 2021). Because this reflects a crucial process in an academic writing task context (Rouet and Britt, 2011; Sonia et al., 2023; Vandermeulen et al., 2023b), not least within higher education, such a writing motivation assessment tool may complement existing measures of writing self-efficacy. Therefore, the main purpose of the current study was to develop a scale targeting the extent to which students are confident they can write an academic text that integrates content from several different sources. In addition, we provided some preliminary validation data for this measure by testing a unidimensional model of the construct in a sample of Norwegian university students, as well as by examining relationships between participants’ scores on this measure and a range of individual difference background and cognitive variables. Specifically, the following four questions guided our research:

1. Are participants’ writing self-efficacy scores based on our measure characterized by a unidimensional structure?
2. Are the background variables of gender orientation, language background, study experience, and previous writing achievement related to participants’ scores on our writing self-efficacy measure?
3. Are the cognitive variables of reading comprehension, working memory, and executive functions related to participants’ scores on this measure?
4. What is the relative contribution of the measured background and cognitive variables to participants’ scores on the writing self-efficacy measure?

Based on the way we designed our writing self-efficacy measure (see the *Method* section), we expected it to be characterized by a unidimensional structure. Regarding the background variables, we did not expect gender orientation or language background to be related to participants’ scores on our measure. This is because prior research has indicated that gender-related differences in writing self-efficacy may be reduced or eliminated at higher educational levels, and because our participants could be expected to be quite proficient in Norwegian although they differed with respect to language background (see *Participants* below). Regarding previous writing achievement, we, based on the assumptions of self-efficacy theory (Bandura, 1997) as well as prior research (Pajares et al., 2007b), expected this background variable to be positively related to our measure of writing self-efficacy. We also expected the background variable of study experience to be positively related to our writing self-efficacy measure because more experience with multiple-source based writing tasks in higher education may increase students’ confidence in their ability to successfully complete such tasks. Regarding the cognitive variables, despite a general lack of prior research, in accordance with Hayes’s (1996) theory of writing, we expected reading comprehension, working memory, and executive functions to be positively related to our writing self-efficacy measure. Finally, regarding the relative contribution of the background and cognitive variables that we measured, we expected previous writing achievement to be the strongest predictor of students’ scores on our measure (Pajares et al., 2007b).

## Method

### Participants

Participants were 136 students at the University of Oslo who were enrolled in programs in education (31.6%), special education (23.5%), arts and humanities (22.1%), social sciences (21.3%), and informatics and mathematics (1.5%).^1^ Sixty-five participants were first-year bachelor students, 36 were second-year bachelor students, and 31 were third-year bachelor students, with only four participants being enrolled in master level programs at the time of data collection. Their overall mean age was 24.07 years (*SD* = 6.41), and 77.2% identified as female, 18.4% as male, and 2.9% as other. Most participants (66.7%) had Norwegian as their sole language background, while 19.1% had another language background, and 14.7% had a mixed language background (i.e., Norwegian and another language). However, 95% of the participants were graduated from a Norwegian high school and all their current university level programs were taught in Norwegian. Participation in the study was voluntary and each participant received a gift card worth approximately USD 20 after the data collection. The collection and handling of the data were in accordance with the Norwegian Personal Data Registers Act and were approved by the Norwegian Social Science Data Services.

### Materials

#### Demographic survey

Participants provided information about their age, gender identification (“with which gender do you identify the most?”), study experience, and language background on a brief demographic survey. With respect to study experience, they used a scale ranging from 1 (bachelor first year) to 5 (master second year),^2^ and with respect to language background, they were asked in which language their parents talked to them when they grew up and responded using the three categories of Norwegian, another language, or Norwegian and another language.

#### Measure of previous academic writing achievement

We assessed participants’ previous academic writing achievement by having them self-report their final high-school grade in written language arts class (i.e., written Norwegian). Those grades were based on the language arts teachers’ running evaluations throughout the final high school year, averaged across various written assessment tests and assignments, with mastery of a range of written academic texts representing different genres emphasized within the national curriculum (e.g., literary essays, argumentative texts; Norwegian Directorate for Education and Training, 2016, 2020). Of note is that Norwegian high-school students engage in multiple-source based writing in different subjects (e.g., language arts and history). Such writing activities are grounded in the national core curriculum, which provides the overarching values and principles for grades 1–13, including critical thinking and the use of different knowledge sources (Norwegian Ministry of Education and Research, 2017). Based on the Norwegian grading system for high school, ranging from 1 (not good) to 6 (excellent), participants rated their previous academic writing achievement on a 6-point scale. Of note is that self-reported grades have been found to correlate highly (approx. 0.90) with the grades provided by the teachers (Dickhäuser and Plenter, 2005; Hofer et al., 2012). Although students’ self-reports may slightly overestimate their actual grades, such overestimation has been found to be unrelated to gender as well as to students’ self-concept and achievement in the domain (Dickhäuser and Plenter, 2005).

#### Measure of reading comprehension

We assessed reading comprehension by means of a Norwegian adaptation of a cloze test developed by Jensen and Elbro (2022), which required readers to draw global, situation level (Kintsch, 1988) inferences in order to fill in each of the gaps. This measure consisted of 34 2-4-sentence passages with one gap in each passage and four alternative words provided for each gap. Correct refilling of the gaps could only be achieved by drawing inferences regarding the global situation described in the passage (i.e., situation model construction; Kintsch, 1988). As an example, an English translation of one passage read:

She had to be ready in two hours so she was in a bit of a rush. The bag was already in the car and the ticket, keys, and wallet were in her pocket. Her husband ran after her with her [*passport*, packed lunch, shopping list, USB key]. It was lucky, otherwise she would not have got very far.

Jensen and Elbro (2022, p. 1233)

The Danish version of this measure was validated by Jensen and Elbro (2022), who demonstrated that the scores of adult readers were highly correlated with their scores on a standardized reading comprehension test as well as with their scores on other reading-relevant measures (vocabulary, sentence comprehension, topic identification). Recently, Salmerón et al. (2022) also provided some preliminary validation data for a Spanish adaptation of this measure.

Participants read the passages and refilled as many gaps as possible during a period of 10 min. Scoring was done by counting the number of correctly refilled gaps (possible maximum score = 34). The internal consistency reliability for participants’ scores on the measure (Cronbach’s α) was 0.84.

#### Measure of working memory

Working memory was measured with a Norwegian adaptation of Swanson and Trahan’s (1992) Working Memory Span Task, which is based on the technique originally developed by Daneman and Carpenter (1980). The Norwegian adaptation has been used and validated in much prior work with postsecondary students (e.g., Delgado et al., 2020; Bråten et al., 2022; Haverkamp and Bråten, 2022). The materials consisted of 42 unrelated declarative sentences, five to 12 words in length, which were organized into 12 sets of sentences. The number of sentences in each set ranged from two to five, and the sentences in each set were read aloud to participants with an interval of two seconds between each sentence. Participants were asked to comprehend the sentences so that they could answer a question about the content of one of the sentences as soon as the final sentence in the set was read. Then, on the same response form, they should write down the final word of each sentence in the set. The working memory task was scored by counting the total number of final words recalled across all 12 sets (possible maximum score = 42) but points were awarded for correctly recalled final words only if the comprehension question for the set was answered correctly. The internal consistency reliability (Cronbach’s α) for participants’ scores on the measure was 0.87.

#### Measure of executive functions

To measure executive functions, we used 19 items from a Norwegian adaptation of the Executive Functions for Learning Inventory (EFLI; Follmer and Tise, 2022) to target participants’ inhibitory and attentional control (10 items), shifting (5 items), and updating (4 items). The items concerning inhibitory and attentional control focused on the ability to deliberately suppress impulsive or dominant responses and devote sustained attention to relevant tasks (sample item: I am good at focusing on what is most relevant to the task I’m working on). The items concerning shifting focused on the ability to switch flexibly and effectively between tasks and activities (sample item: I can move back and forth between tasks to finish what I have started). The items concerning updating focused on the ability to monitor and update (add/delete) working memory content as required by a task (sample item: I can juggle multiple things at the same time in my mind). Each item was rated on a 5-point scale ranging from *fits very poorly* (1) to *fits very well* (5). In terms of validity, Follmer and Tise (2022) showed that scores on the EFLI both indirectly (via cross-text elaboration strategies) and directly predicted multiple text comprehension in a sample of American college students and actually were a better predictor in this regard than a direct (i.e., task-based) measure of executive functions.

In the current study, a confirmatory factor analysis (CFA) with the lavan R package (R Core Team, 2020) did not support a three-dimensional structure in which each of the 19 items loaded on its designated factor. However, after removing five items with low loadings (< 0.50) and including four correlations between residuals that were suggested by the modification indices and seemed methodologically as well as substantially justified, the re-specified model had an acceptable fit to the data, with χ^2^(70) = 109.01, *p* = 0.002; confirmatory fit index (CFI) = 0.95; root mean square error of approximation (RMSEA) = 0.064, 90% CI (0.039–0.087); and standardized root mean square residual (SRMR) = 0.061. The internal consistency reliability (Cronbach’s α) for participants’ scores on the seven items measuring inhibitory and attentional control was 0.83. For their scores on the three items measuring shifting, it was 0.70, and for their scores on the four items measuring updating, it was 0.75.

#### Measure of multiple-source based academic writing self-efficacy

To assess participant’s confidence in their ability to write an academic text or paper that integrates or synthesizes content from multiple textual sources, we developed the Multiple-Source Based Academic Writing Self-Efficacy Scale (MAWSES). The 8-item MAWSES was based on Bandura’s (1997) conceptualization of self-efficacy applied to the specific writing process of integrating information across multiple sources and to the specific writing task of composing an academic text. Thus, this scale can be considered to target “process-focused writing self-efficacy” (Abdel Latif, 2021, p. 13) by focusing on writers’ confidence in their ability to perform the writing process of cross-source integration. At the same time, however, it can be considered task-specific by focusing on the specific task of producing an academic text or paper. Taken together, this means that the MAWSES can be considered an integration process for academic text self-efficacy measure.

As no prior writing self-efficacy measure to the best of our knowledge focused on this particular process within academic writing (for review of existing writing self-efficacy measures, see Abdel Latif, 2021), we consulted the literature on synthesis writing (Spivey and King, 1989; Segev-Miller, 2007; Solé et al., 2013; Vandermeulen et al., 2020a,b) as well as on written task products used for comprehension assessment within multiple document literacy (e.g., Ferguson and Bråten, 2013; Barzilai and Ka’adan, 2017; Du and List, 2020; McCarthy et al., 2022; Kullberg et al., 2023) in developing the items for our measure. In brief, these items were developed to represent a core process in writing synthesis texts and communicating an integrated understanding based on multiple source reading, with different aspects of this process, such as dealing with inconsistencies, explaining similarities and differences between perspectives, creating overview and comprehensiveness, and producing a new, original text, presumably captured by the items.

Participants were asked to evaluate their own ability to write academic texts by rating each item on a 10-point scale ranging from *quite confident that I cannot perform this* (1) to *quite confident that I can perform this* (10). All items on the MAWSES are displayed in Table 1 together with descriptive information for each item. Descriptive information for the entire measure and the reliability of participants’ scores are also included in the Results section.

**Table 1:** Descriptive statistics and factor loadings for the items of the multiple-source based academic writing self-efficacy scale.

Item	Item no.	*M*	*SD*	Skewness	Kurtosis	Loading
I can write an academic text that integrates content from several different sources	1	8.25	1.88	−1.10	1.23	0.77
I can combine information from several different texts I have read and write a new, original academic text based on these texts	2	7.30	2.15	−0.83	0.44	0.74
When the content of the texts I have read is inconsistent, I can still write a coherent academic text based on them	3	6.99	2.16	−0.68	−0.03	0.84
When I write academic texts, I can present a complete picture of a topic based on various academic texts I have read about it	4	7.66	1.76	−0.55	−0.31	0.80
I can explain a complex topic in a clear and understandable way when I write academic texts based on several different source texts	5	6.76	1.97	−0.45	−0.25	0.81
When I write academic texts, I can evaluate and integrate different arguments about an issue that are presented in the texts I have read about it	6	7.50	1.99	−0.71	0.11	0.87
When I write academic texts based on different sources, I can structure the text such that it becomes easy for the reader to understand what I write	7	7.46	1.85	−0.79	0.50	0.68
I can explain differences and similarities between different perspectives when I write academic texts based on multiple sources	8	7.80	1.76	1.00	1.53	0.86

### Procedure

The second and third authors collected all the data during individual 60-min sessions in a quiet room at the university. The working memory measure was administered orally before participants completed the demographic survey, the Multiple-Source Based Academic Writing Self-Efficacy Scale (MAWSES), and the reading comprehension measure independently on paper. Finally, participants completed the inventory of executive functions targeting inhibitory and attentional control, shifting, and updating and the measure of previous academic writing achievement using a web based questionnaire accessible through a link on a laptop computer.

### Data analysis

To examine the construct validity of the MAWSES, we first analyzed all item scores descriptively and then performed a CFA by means of the lavan R package (R Core Team, 2020) to test how well a unidimensional model fit the data. We used chi-square statistics as well as the fit indices of CFI, RMSEA, and SRMR to evaluate the fit of the unidimensional model. Based on proposed cut-off criteria for the evaluation of the goodness of fit (Hu and Bentler, 1999; Marsh et al., 2004; Brown, 2015), we adopted the following criteria for good model fit: CFI ≥ 0.95, RMSEA ≤0.06, and SRMR ≤0.06. In addition to the overall model fit, we examined the factor loadings and the internal consistency reliability of participants’ MAWSES scores.

Further, we used one-way between-subjects analyses of variance (ANOVAs) to examine whether participants who differed with respect to gender identification and language background, respectively, scored differently on the MAWSES, and we conducted a correlational analysis to examine zero-order correlations (Pearson’s *r*) between participants’ scores on the MAWSES and their scores on the variables of study experience, previous academic writing achievement, reading comprehension, and executive functions (i.e., inhibitory and attentional control, shifting, and updating).

Finally, based on the resulting correlational pattern, we conducted a simultaneous multiple regression analysis to examine the relative contribution of participants’ study experience, previous academic writing achievement, reading comprehension, and executive functions to their multiple-source based academic writing self-efficacy.

## Results

As can be seen in Table 1, our examination of the distributional properties of the item-level MAWSES variables showed that all items were approximately normally distributed, with only one item having a skewness value slightly below 1 (−1.10) and only two items having kurtosis values slightly above 1 (1.23, 1.53). Ordinary maximum likelihood extraction was therefore used for the CFA.

The unidimensional model of multiple-source based academic writing self-efficacy that we specified and tested by means of CFA had an acceptable fit to the data, with χ^2^(20) = 39.54, *p* = 0.006; CFI = 0.98; RMSEA = 0.085, 90% CI (0.045–0.123); SRMR = 0.033, with factor loadings ranging from 0.70 to 0.87. However, the RMSEA was somewhat higher than desirable and the modification indices indicated that the fit could be improved by allowing the error variances of items 5 (I can explain a complex topic in a clear and understandable way when I write academic texts based on several different source texts) and 7 (When I write academic texts based on different sources, I can structure the text such that it becomes easy for the reader to understand what I write) to correlate. Because these items to some extent were similarly worded (understandable/easy for the reader to understand) and because both may seem to capture some kind of audience awareness among writers, we considered it both methodologically and substantially justifiable to re-specify the model with their errors freed to correlate. The re-specified model fit the data well, with χ^2^(19) = 28.59, *p* = 0.073; CFI = 0.99; TLI = 0.98; RMSEA = 0.061, 90% CI (0.000–0.104); SRMR = 0.027. The re-specification resulted in a statistically significantly improvement of the model fit, with ∆χ^2^(1) = 10.95, *p* < 0.001. The loadings of the eight items ranged from 0.68 to 0.87 (see Table 1), and the standardized estimate of the correlated error was 0.303. The internal consistency reliability of participants’ MAWSES scores was high (Cronbach’s α = 0.93).

Further, one-way between-subjects ANOVAs showed that gender identification (female: *M* = 7.49, *SD* = 1.66; male: *M* = 7.39, *SD* = 1.28) or language background (Norwegian: *M* = 7.57, *SD* = 1.50; another language: *M* = 7.04, *SD* = 1.97; Norwegian and another language: *M* = 7.56, *SD* = 1.50) did not matter in terms of participants’ MAWSES scores, with *F* (1, 128) = 0.08, *p* = 0.778, for gender identification, and *F* (2, 133) = 1.15, *p* = 0.319, for language background. However, a correlational analysis showed that participants’ scores on the MAWSES were positively and statistically significantly correlated with their study experience (*r* = 0.203, *p* = 0.019), indicating higher writing self-efficacy the longer participants had studied at bachelor level, as well as with their previous academic writing achievement (*r* = 0.343, *p* < 0.001). Further, participants’ MAWSES scores were positively and statistically significantly correlated with reading comprehension (*r* = 0.211, *p* = 0.014) and the three types of executive functions that we measured (inhibitory and attentional control: *r* = 0.253, *p* = 0.003; shifting: *r* = 0.202, *p* = 0.019; updating: *r* = 0.333, *p* < 0.001), but not with working memory (*r* = 0.135, *p* = 0.119). Results of the correlational analysis are shown in Table 2, which also includes descriptive information (*M*, *SD*, skewness, and kurtosis) about the variables.

**Table 2:** Descriptive statistics and zero-order correlations for measured variables.

Variables	1	2	3	4	5	6	7	8
1. Study experience	–							
2. Previous writing achievement	0.152	–						
3. Reading comprehension	0.160	0.348^***^	–					
4. Working memory	−0.012	0.204^*^	0.410^***^	–				
5. Inhibitory and attentional control	0.144	0.273^**^	0.052	0.048	–			
6. Shifting	0.067	0.002	0.004	−0.007	0.419^***^	–		
7. Updating	0.097	0.133	0.060	0.067	0.439^***^	0.464^***^	–	
8. Writing self-efficacy (MAWSES)	0.203^*^	0.343^***^	0.211^*^	0.135	0.253^**^	0.202^*^	0.333^***^	–
*M*	1.74	4.69	24.87	20.66	3.30	3.45	3.27	7.47
*SD*	0.82	0.84	5.03	8.16	0.73	0.79	0.78	1.60
Skewness	0.51	−0.26	−0.95	0.10	−0.17	−0.51	0.16	−0.80
Kurtosis	−1.31	−0.03	0.97	−0.49	−0.55	−0.09	−0.63	0.83

^*^p < 0.05, ^**^p < 0.01, ^***^p < 0.001. Study experience is scored 1, 2, or 3 depending on bachelor program level (first, second, or third year).

**Figure 1:**
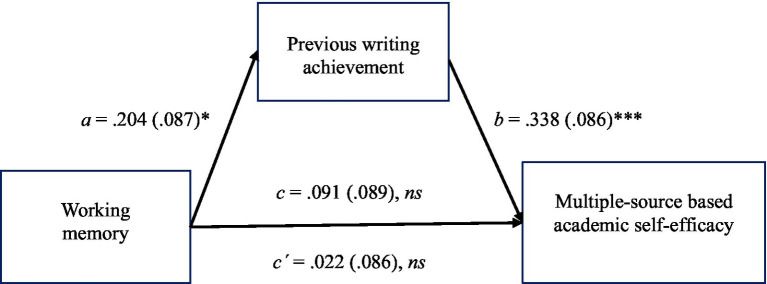
Mediation model for the effect of working memory on multiple-source based writing self-efficacy (MAWSES) with previous writing achievement as a mediator (standardized coefficients). ^*^p < 0.05, ^***^p < 0.001.

Although working memory was not statistically significantly related to writing self-efficacy, we also performed an exploratory mediation analysis to probe if there was an indirect relationship between working memory and the MAWSES scores via previous writing achievement. In doing this, we used the bootstrapping approach available in the PROCESS Procedure for SPSS Version 4.0 (Hayes, 2022), which holds no assumption about the statistical significance of the *c* path. The indirect relationship was tested with a bootstrap estimation approach with 5,000 samples. The results of the mediation analysis are shown in Figure 1.

There was a positive statistically significant indirect relationship between working memory and multiple-source based writing self-efficacy via previous writing achievement, with an estimate of 0.069 (CI_95%_: 0.016–0.135). Working memory was a statistically significant predictor of previous writing achievement (*b* = 0.204, *SE* = 0.087, *p* = 0.021), which, in turn, was a statistically significant predictor of writing self-efficacy (*b* = 0.338, *SE* = 0.086, *p* = 0.0001). Consistent with a full mediation, the direct relationship between working memory and writing self-efficacy remained statistically non-significant (*b* = 0.022, *SE* = 0.086, *p* = 0.795). The model explained 12% of the variance, *R*^2^ = 0.12, *F* (2, 125) = 8.33, *p* = 0.0004.

Finally, based on the zero-order correlations, we performed a simultaneous multiple regression analysis to examine the contribution of participants’ study experience, previous writing achievement, reading comprehension, and the executive functions of inhibitory and attentional control, shifting, and updating to their MAWSES scores. Although the positive correlations between the three executive function measures ranged from 0.419 to 0.464 (see Table 2), shared variances from 17.6 to 21.5% indicated that three distinct executive function constructs actually were captured by these measures. We therefore decided to keep all three measures in the equation. A simultaneous multiple regression analysis was performed in this study because we wanted to examine the relative contribution of the predictors, including the three executive function constructs, to multiple-source based writing self-efficacy. The results of this analysis are displayed in Table 3. Taken together, the six predictors explained 24% of the variance in MAWSES scores, *F* (6, 121) = 11.12, *p* < 0.001. The variables that uniquely and positively predicted multiple-source based writing self-efficacy in this analysis were previous writing achievement (β = 0.24, *p* = 0.009) and the executive function of updating (β = 0.24, *p* = 0.013).

**Table 3:** Results of multiple regression analysis for variables predicting multiple-source based academic writing self-efficacy.

Predictor	*B*	*SE B*	β
Study experience	0.24	0.15	0.13
Previous writing achievement	0.43	0.16	0.24^**^
Reading comprehension	0.03	0.03	0.10
Inhibitory and attentional control	0.11	0.20	0.06
Shifting	0.07	0.18	0.04
Updating	0.46	0.18	0.24^*^

^*^p < 0.05, ^**^p < 0.01.

## Discussion

Writers’ confidence in their ability to write an academic text or paper that integrates or synthesizes content from multiple sources is an important aspect of writing motivation across educational levels. In the current study, we developed a measure targeting this particular form of writing motivation, which we called the MAWSES, and analyzed the structure of the scores on this measure by means of confirmatory factor analysis as well as the relationships between the resulting construct and a range of relevant individual difference background and cognitive variables. In this way, we essentially followed the classic procedure for construct validation described by Cronbach and colleagues (Cronbach and Meehl, 1955; Cronbach, 1990).

First, the confirmatory factor analysis indicated that the scores on the multiple-source based academic writing self-efficacy measure that we developed could be characterized by a unidimensional structure.

Second, although participants’ scores on our measure did not differ by gender orientation or language background, they correlated positively with the background variables of study experience and previous writing achievement. Regarding gender orientation, this finding is consistent with prior research indicating that gender-related differences in writing self-efficacy may disappear at higher educational levels (Abdel Latif, 2021), and regarding language background, our finding suggests that participants having another language background than Norwegian or a mixed language background did not perceive their current language ability as problematic (Abdel Latif, 2021). Relevant in this regard is the fact that the vast majority of the participants, irrespective of language background, were graduated from a Norwegian high school and that their university programs also were taught in Norwegian. The positive relationship found between study experience and participants’ scores on the MAWSES suggests that more extensive study experience beyond high school may lead to higher writing self-efficacy (Mitchell et al., 2021), possibly because many writing assignments requiring integration of multiple sources followed by supportive feedback may increase students’ perceived self-efficacy for performing such tasks (Bruning and Horn, 2000). The positive relationship found between prior writing achievement and participants’ MAWSES scores is consistent with Bandura’s (1997) theory of self-efficacy as well as with prior research on the antescedents of students’ writing self-efficacy (Pajares et al., 2007b).

Third, among the cognitive variables, reading comprehension and the executive functions of inhibitory and attentional control, shifting, and updating were all positively related to participants’ MAWSES scores, and working memory was indirectly related to those scores via previous writing achievement. These findings are consistent with Hayes’s (1996) conceptualization of relationships between reading comprehension (termed “text interpretation” by Hayes), executive functions, and writing motivation. It also stands to reason that working memory capacity may underlie students’ history of achievement in the domain of writing, which, in turn, contributes to their multiple-source based academic writing self-efficacy.

Fourth, when examining the relative contribution of the individual difference variables that were positively correlated with the writing self-efficacy scores, previous writing achievement and updating emerged as the strongest predictors. Regarding previous writing achievement, this finding is consistent with prior research comparing successful performance in the domain to other potential sources of writing self-efficacy (Pajares et al., 2007b). Further, the fact that updating was a relatively strong predictor in this multivariate context may suggest that the ability to continuously monitor and add/delete working memory content may serve processes of writing such as controlling the relevance/irrelevance of content retrieved from long-term memory (Miyake and Friedman, 2012) and thereby boost students’ perceived self-efficacy for mastering multiple-source based writing tasks.

Taken together, our findings provide preliminary evidence suggesting that the MAWSES is a reliable and valid measure of an important aspect of writing motivation in the contexts of multiple document literacy and synthesis writing. As a unitary construct, students’ confidence in their ability to accomplish multiple-source based, integrated academic writing tasks was associated with their university level study experience and their previous writing achievement, as well as directly with their reading comprehension and executive functions and indirectly with their working memory capacity. Such relationships are consistent with theories of self-efficacy (Bandura, 1997) and writing (Hayes, 1996; MacArthur and Graham, 2016; Graham, 2018; McNamara and Allen, 2018), as well as with prior writing motivation research (Abdel Latif, 2021).

One limitation of the current validation effort is that we studied participants’ scores on the MAWSES in relation to other variables that can be considered antecedents of the construct rather than its consequences, with further validation research needed to examine the predictability of MAWSES for multiple-source based, integrated academic writing performance with other relevant predictors controlled for. That said, it should also be noted that prior writing motivation research, including research on writing self-efficacy, hitherto seems to have been more concerned about the consequences of writing motivation than about its antecedents (Abdel Latif, 2021). Of course, our findings are also limited by the particular sample that we included and by the way we measured the variables in question, with further research needed to probe the generalizability of these findings across student populations and measures. For example, future research should try to replicate our findings with other measures of previous writing achievement than the self-reports of final high-school grades that we used in this study. In particular, more direct and proximal measures of previous writing achievement should be used in future testing of the indirect relationship between working memory and multiple-source based writing self-efficacy via previous writing achievement that we explored in this study. Regarding the writing self-efficacy measure that we developed, it also seems pertinent to adapt the items to writing within specific academic domains as well as to writing about specific topics within those domains. In addition, the specificity of measurement may be further increased by adapting the items to multiple-source based integrated writing for different academic task purposes (e.g., summary writing in order to learn, cross-text elaboration in order to demonstrate understanding, argumentative writing in order to persuade or reach a balanced conclusion; Nussbaum, 2008). Finally, other theoretically grounded writing motivation constructs, such as writing task values and writing goal orientations (Graham, 2018), should be adapted to multiple-source based academic writing in future research.

Despite the limitations of the current study, we remain optimistic about the potential applications of the writing motivation measure we created. Beyond the potential of the measured writing self-efficacy construct to predict both processes and products of integrated academic writing is its potential to moderate or mediate the effects of interventions targeting integrated academic writing, assess the motivational outcome of such interventions, and provide information about students’ writing motivation trajectories within and across educational levels. For example, efforts to improve students’ multiple-source based writing in academic contexts might be differentially successful depending on how confident students are they can complete such challenging writing tasks, with the writing motivation measure we created serving as a tool in examining potentially moderated effects of writing interventions. Further, when writing researchers try to assess the motivational effects of instruction in multiple-source based writing (MacArthur et al., 2023), the MAWSES may be a more sensitive measure of such effects compared to motivation measures that do not target this particular type of academic writing. Finally, this measure may be used to study the development of writing motivation in different academic programs within higher education, as well as contextual influences on motivational development in this regard.

## Data availability statement

The raw data supporting the conclusions of this article will be made available by the authors, without undue reservation.

## Ethics statement

The studies involving human participants were reviewed and approved by The Norwegian Social Science Data Services. The patients/participants provided their written informed consent to participate in this study.

## Author contributions

IB: conceptualization, methodology, analysis, writing, and supervision. YH: conceptualization, methodology, investigation, analysis, and writing. NL: conceptualization, methodology, and investigation. HS: conceptualization, methodology, and reviewing. All authors contributed to the article and approved the submitted version.

## Conflict of interest

The authors declare that the research was conducted in the absence of any commercial or financial relationships that could be construed as a potential conflict of interest.

## Publisher’s note

All claims expressed in this article are solely those of the authors and do not necessarily represent those of their affiliated organizations, or those of the publisher, the editors and the reviewers. Any product that may be evaluated in this article, or claim that may be made by its manufacturer, is not guaranteed or endorsed by the publisher.

## Footnotes

^1^This study is part of a larger project. However, research questions, materials, analyses, and results are unique to this study and not reported elsewhere.

^2^A bachelor’s degree at the University of Oslo is normally completed in three years, with a master’s degree normally requiring two additional years.
